# Legume Overseeding and P Fertilization Increases Microbial Activity and Decreases the Relative Abundance of AM Fungi in Pampas Natural Pastures

**DOI:** 10.3390/microorganisms11061383

**Published:** 2023-05-24

**Authors:** Gastón Azziz, Cristina Frade, José M. Igual, Amabelia del Pino, Felipe Lezama, Ángel Valverde

**Affiliations:** 1 Laboratorio de Microbiología, Departamento de Biología Vegetal, Facultad de Agronomía, Universidad de la República, Montevideo 12900, Uruguay; gazziz@fagro.edu.uy; 2 Grupo de Interacción Planta-Microorganismo, Instituto de Recursos Naturales y Agrobiología de Salamanca, CSIC, 37008 Salamanca, Spain; cristina.frade@irnasa.csic.es; 3 Departamento de Suelos y Aguas, Facultad de Agronomía, Universidad de la República, Montevideo 12900, Uruguay; amabelia@fagro.edu.uy; 4 Departamento de Sistemas Ambientales, Facultad de Agronomía, Universidad de la República, Montevideo 12900, Uruguay; flezama@fagro.edu.uy

**Keywords:** extracellular enzyme activities, grasslands, grazing, Illumina sequencing, legumes, livestock, phosphorus fertilization, PLFAs, soil microbiology

## Abstract

Natural grasslands provide a valuable resource for livestock grazing. In many parts of South America, legume overseeding and P fertilization are commonly used to enhance primary productivity. The effect of this practice on the plant community is well established. However, how this management regime affects the soil microbiome is less known. Here, to contribute to filling this knowledge gap, we analyzed the effect of *Lotus subbiflorus* overseeding, together with P fertilization, on soil microbial community diversity and activity in the Uruguayan Pampa region. The results showed that plant communities in the natural grassland paddocks significantly differed from those of the managed paddocks. In contrast, neither microbial biomass and respiration nor microbial diversity was significantly affected by management, although the structure of the bacterial and fungal communities were correlated with those of the plant communities. AM Fungi relative abundance, as well as several enzyme activities, were significantly affected by management. This could have consequences for the C, N, and P content of SOM in these soils, which in turn might affect SOM degradation.

## 1. Introduction

Grasslands cover about 40% of the Earth’s surface and 69% of the Earth’s agricultural land area ([1] and references therein). They provide many ecosystem services such as livestock production, carbon storage, pollination, and they also regulate climate, soil erosion, and water retention [2].

South American temperate grasslands (i.e., paramos, puna, Pampas and campos, and the Patagonian steppe) have sustained livestock production since the introduction of cattle and sheep by European colonizers [3]. Traditionally, this production has been accomplished with little intervention and minimal input [4]. However, to compete with other land use activities, such as arable farming and forestry [5,6], and produce more meat and milk to meet increasing world demands [7], there is a need to increase the productivity of grasslands. Thus, in some areas of the Pampas, as well as in other grasslands of the world, legume overseeding in combination with phosphorus fertilization (hereafter LP) has been applied [8]. A sufficient supply of inorganic phosphorus is required for nodule development and symbiotic N-fixation in legumes [9]. Legume overseeding generally increases short-term forage production, while avoiding the total replacement of the original natural grassland cover. Other benefits of this practice include reduced dependence on fossil energy and industrial N-fertilizer, lower quantities of harmful emissions of greenhouse gases and nitrate, and lower production costs [10].

The effect of legume overseeding on grassland plant communities has been extensively studied. For instance, Jaurena et al. [11] reported marked changes in plant diversity and composition after 20 years of legume overseeding and different amounts of P fertilization. However, much less is known about the impact of LP on soil microbial communities, particularly in South American grasslands.

Soil microbes are important regulators of plant diversity and productivity [12]. For example, bacteria known as rhizobia are able to fix nitrogen in symbiosis with legumes. Legumes such as *Lotus* spp. Can establish symbiotic relationships with nearly 20 species of five rhizobia genera within the families *Phyllobacteriaceae*, *Rhizobiaceae*, and *Nitrobacteraceae* (Alphaproteobacteria class) [13]. Phosphorous acquisition by plants is also partially mediated by the action of soil microorganisms, particularly phosphorus acquiring arbuscular mycorrhiza fungi (AMF) and phosphate solubilizing bacteria (PSB). Most P in soil is in organic form and therefore needs to be mineralized by phosphatase enzymes before being available to plants. Alkaline phosphatases, specifically those encoded by the *phoD* gene, have been found in diverse environments and are among the most studied functional genes in microbial soil diversity surveys [14].

Plants interact with the soil microbial community mainly through root exudates, which typically comprise primary metabolites such as sugars, amino acids, and carboxylic acids, as well as a diverse set of secondary metabolites [15]. Root exudate compounds have a multitude of effects on rhizosphere microbes by acting as signaling molecules, attractants, stimulants, but also as inhibitors or repellents [16]. The amount and composition of the root exudates depend on a number of factors, including plant species, growth stage, nutrient availability, and the interaction with other plants and microorganisms [17]. Most of the few studies that have investigated the effect of legume overseeding on soil microbial communities have done so in experimental systems with low plant diversity [18,19], or have used experimental settings that may not match farmers’ practices [20].

Here, we investigated in Pampas grasslands the effect of LP on soil microbial diversity and composition (using next generation sequencing and PLFAs) and functioning (by means of extracellular enzyme activities and microbial respiration). We hypothesize that (1) LP, by selecting specific microbes, decreases soil microbial diversity and shapes microbial community composition, that (2) LP increases the abundance of nutrients entering the soil (through plant litter and root exudates), which increases microbial activity, and that (3) these changes are associated with plant diversity and composition.

## 2. Materials and Methods

### 2.1. Sampling Sites Description and Soil Collection

The five study sites, selected based on a previous work [21] and distant between ca. 0.7 and 23 km, were located in the east-center zone of Uruguay. The sites are privately owned and the fields are used for cattle and sheep grazing. At each site we sampled two paired plots, one under natural grassland (NG) and the other subjected to legume overseeding with *Lotus subbiflorus* and fertilized with phosphorous (NGLP). The plots of each pair are separated only by a wire fence. Thus, there were five replicates per treatment (NG and NGLP) paired by study site. The main characteristics of the soils are detailed in the Table S1. Soils from the sites are characterized as Alfisols and Mollisols and have a sandy loam texture in the A horizon.

Soil and vegetation sampling was conducted in the spring of 2021. Fifteen soil cores 2.5 cm wide and 10 cm depth were randomly collected from each plot and mixed together in plastic bags, resulting in a composite sample for each plot (5 sites × 2 plots (NG and NGLP) = 10 soil samples). After collection, samples were transported to the laboratory and stored at 4 °C until processed within 3 days. Soils were sieved through a 2 mm mesh and aliquots of 80 g were lyophilized for 48 h and sent to the IRNASA (Instituto de Recursos Naturales y Agrobiología de Salamanca, Salamanca, Spain), for PLFAs and extracellular enzymes activity analysis.

### 2.2. Vegetation Sampling

At each plot, plant composition was recorded by six 1 m^2^ quadrats systematically placed with respect to the fence dividing the treatments (and at least 5 m from the fence avoiding edge effect). The quadrats were separated by three meters from each other. In each quadrat we recorded all vascular plant species and visually estimated the percentage aerial cover of each individual species following the Braun-Blanquet abundance scale for values under 5% and in steps of 5% for higher values. Relative abundance matrixes were obtained from averaging the six squares of each plot.

### 2.3. DNA Extraction and Illumina Amplicon Sequencing

Soil DNA was extracted from fresh soil using the DNeasy PowerSoil Pro Kit (QIAGEN; Barcelona, Spain), following the manufacturer’s instructions. The integrity of the extracted DNA was checked using a 0.8% (*w*/*v*) agarose gel electrophoresis in TBE buffer. The quantity and quality of the DNA were estimated by UV spectrophotometry using a NanoDrop (Thermo Fisher Scientific; Madrid, Spain), reading the absorbance at 260 nm and the 260/280 ratio, respectively.

Bacterial community composition was determined by sequencing the V4 region of the 16S rRNA gene using the primers 515F (GTGYCAGCMGCCGCGGTAA) and 806R (GGACTACNVGGGTWTCTAAT) [22]. Fungal communities were assessed by sequencing the internal transcriber spacer (ITS1) region using the primers ITS1F (CTTGGTCATTTAGAGGAAGTAA) and ITS2R (GCTGCGTTCTTCATCGATGC) [22]. The *phoD* genes, which code for an alkaline phosphatase, were obtained using the primers phoD-F733 (TGGGAYGATCAYGARGT) and phoD-R1083 (CTGSGCSAKSACRTTCCA) [23].

Sequencing was carried out at Molecular Research LP, Shallowater, USA. The sequencing method used was the bacterial tag encoded FLX amplicon sequencing (bTEFAP) carried out in a MiSeq^TM^ system by Illumina.

The quality of the reads obtained was examined using FastQC [24]. For 16S rRNA genes and ITS, reads were processed using the dada2 package in RStudio [25] to obtain amplicon sequence variants (ASVs). The processing included removal of primers, filtering of low-quality sequences, and trimming of sequences to avoid low quality ends. Sequences were dereplicated before the paired sequences were merged. Finally, chimeras were removed by the consensus method. Reads of the phoD genes were clustered by CD-HIT [26] with parameters “-c 0.95 -n 10 -G 0 -aS 0.9”. Representative sequences for each cluster were classified by alignment against the *phoD* database at FunGene (http://fungene.cme.msu.edu/index.spr, accessed on 19 September 2022) using BLASTn (default parameters). Result hits >1 × 10^−5^ evalue, >80% identity, and >70% alignment coverage were used for species assignment. For the genes with multiple best-hits, the species with the highest frequency and the highest average similarity was defined as the species annotation of the gene. For the 16S rRNA and ITS data taxonomy was assigned to the ASVs using the SILVA (version 138.1) and the UNITE (version 9.0) databases, respectively. Data were rarefied to even depth between samples. After rarefaction, the total number of reads per sample was: 125,409 for the 16S rRNA data, 42,610 for the ITS data, and 8010 for the *phoD* data. The data derived from the dada2 pipeline were used to build a phyloseq object [27].

Alpha diversity indexes, as well as abundance tables at different taxonomic levels, were calculated using the microeco package vs 0.17.0 [28] in RStudio. The microbiome package [29] was used to transform the phyloseq objects to relative abundances values. Microbial functions derived from the 16S rRNA and ITS data were inferred with the *transf_func* command of the microeco package using the FAPROTAX [30] and FUNGuild [31] databases, respectively.

### 2.4. Phospholipid Fatty Acids Profiles Determination

Liophilized soils were used to extract phospholipid fatty acids (PLFAs). Briefly, PLFAs were extracted from 2 g of soils per sample with one-phase chloroform–methanol–phosphate buffer as solvent. Methodology described by Buyer and Sasser [32] was employed to separate non polar lipids from phospholipids; the latter ones were converted to fatty acid methyl esters (FAMEs). Chromatograms were obtained with gas chromatography using an Agilent 7890A GC system (Agilent Technologies, Wilmington, NC, USA) equipped with a flame ionization detector and a 25-m Ultra 2 (5%-phenyl)-methylpolysiloxane column (J&W Scientifc, Folsom, CA, USA). The PLFAD1 method of Sherlock software v6.3 (MIDI Inc., Newark, NJ, USA) was used to identify and quantify the FAMEs. The internal standard 19:0 phosphatidylcholine (Avanti Polar Lipids, Alabaster, AL, USA) was used for the quantification of the FAMEs. Total microbial biomass was calculated as the sum of every single PLFA in each sample and reported as nanomoles of PLFAs per gram of soil. Specific PLFAs were used as biomarkers to quantify biomasses of broad taxonomic microbial groups, according to their characteristic fatty acids: eukaryote, Gram negative and Gram positive bacteria, Actinobacteria, saprophytic fungi, and arbuscular mycorrhizal fungi (AMF) [33]. The sum of cyclopropyl fatty acids and the sum of their monoenoic precursors (cy17:0 + cy19:0)/(16:1ω7 + 18:1ω7) (hereafter G- cy/pre) were calculated as proxies of physiological or nutritional stress in the bacterial communities [34,35].

### 2.5. Extracellular Enzyme Activities

The potential activities of seven extracellular enzymes (EEA) were determined in each soil sample. The fluorimetric method described in Bell et al. [36] was used for the preparation and incubation of the samples. The enzymes whose activities were determined were: α-1,4-glucosidase (AG), β-1,4-glucosidase (BG), β-D-cellobiohydrolase (CB), β-xylosidase (XYL), β-1,4-N-acetylglucosaminidase (NAG), L-leucine aminopeptidase (LAP), and acid phosphatase (PHO). An aliquot (2.75 g) of liophilized soil was rehydrated up to field moist with ultrapure sterile water. Rehydrated soils were then suspended in 91 µL of 50 mM acetate buffer at a pH adjusted with glacial acetic acid to match the soil pH. Soil solutions were homogenized with a domestic blender. In 96 deep well microplates, 800 µL of soil slurries were mixed with 200 µL of 200 µM water solutions of the corresponding flourimetric substrates. Microplates were incubated at 25 °C for 4 h in the dark. After incubation, the microplates were centrifuged (5 min at 2100 g) and 250 µL of supernatant transferred to black ninety-six-well microplates for fluorescence quantification. Fluorescence was read at 365 nm excitation and 450 nm emission in a FLUOstar Omega microplate reader (BMG LABTECH, Ortenberg, Germany). Enzyme activity was calculated from the fluorescence data using a lineal regression according to the method described by Bell et al. [36].

### 2.6. Microbial Respiration

Microbial respiration was determined using the MicroResp^TM^ system (Aberdeen, UK) following the colorimetric method [37]. Lyophilized soil was rehydrated with sterile ultrapure water and incubated at room temperature for 5 days prior to analysis. Briefly, 0.4 g soil samples were weighed into 1.2 mL deep-wells in ninety-six-well microplates (Fisher Scientific). Following the MicroResp^TM^ manual, microplates containing soil were placed over detector plates and incubated at 25 °C. Detector plates for CO_2_ were prepared as described by Campbell et al. (2003) [37], and the absorbance of the detector plate wells was quantified at 570 nm in a ThermoScientific™ Multiskan™ GO Microplate Spectrophotometer (Thermo Fisher Scientific, Madrid, Spain) at time 0 and after 24 h of incubation. The conversion from absorbance to CO_2_-C (µg g^−1^ h^−1^) was performed using a calibration curve prepared from plate wells in closed bottles with a range of CO_2_ concentrations.

### 2.7. Soil Physicochemical Properties

After drying at 40 °C the soil samples were crushed to pass a 2 mm mesh. The content of available P was determined according to Bray 1 method [38]. Soil pH was determined in a 1:2.5 soil: water suspension. Exchangeable acidity was analyzed according to Mc Lean [39]. The organic C content was analyzed by wet oxidation with potassium dichromate [40]. Exchangeable basic cations were extracted with neutral ammonium acetate and determined by emission (K and Na) and absorption atomic spectrometry (Ca and Mg) [41].

### 2.8. Statistical Analysis

All statistical analyses were completed in R version 4.2.2 [42]. Diversity indexes, as well as relative abundances of specific microbial taxa, were compared between NG and NGLP treatments using paired *t*-test. Data were checked for normality and homoscedasticity with the Shapiro–Wilk and Levene’s tests, respectively. The ASVs/OTUs relative abundance matrixes were used to compare the population structure of each microbial group in all samples. Plant and microbial population structures were visualized using non-metric multidimensional scaling (NMDS) based on Bray–Curtis distances. The differences between populations that arise due to treatment were tested using PERMANOVA function “adonis2” in the R package Vegan [43]. The basic R function coefficients were used to extract the top explaining variables of the PERMANOVA results.

## 3. Results

### 3.1. Effect of LP on Plant Diversity and Composition

Plant communities were dominated by grasses of the class Liliopsida (67.1% cover on average) and members of the Magnoliopsida (25.6%) (Figure S1). Considering all samples, a total of 36 plant families were found, of which 28 belonged to Magnoliopsida, 7 to Liliopsida and one to Psilotopsida. The most abundant families were *Poaceae* (55.8%) and *Fabaceae* (9.5%) (Figure S2). The total number of genera found was 101; 56 genera belonged to Magnoliopsida, 44 to Liliopsida, and 1 to Psilotopsida. The expected presence of *Lotus* in the NGLP treatment was confirmed, although it was not evenly abundant in the different sites (Figure S3).

**Figure 1:**
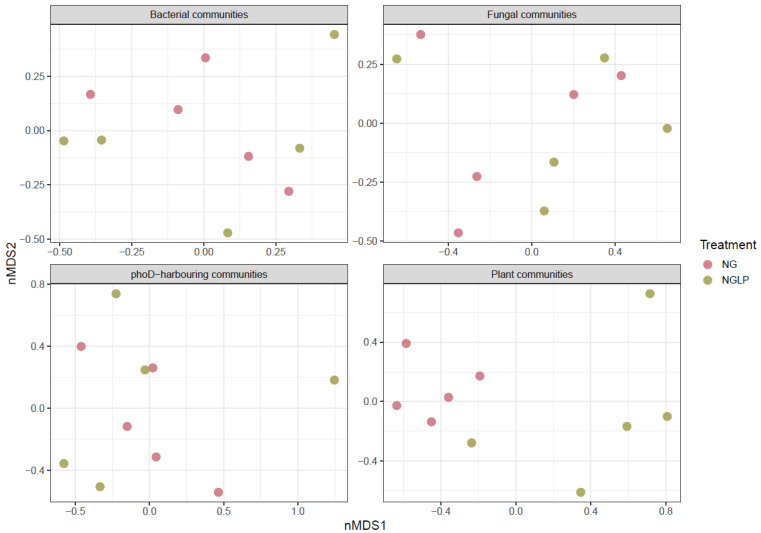
Non-metric multidimensional scaling (NMDS) ordination of the bacterial, fungal, *phoD*-harbouring, and plant communities. NG = natural grassland, NGLP = natural grassland overseeded with *Lotus subbiflorus* and fertilized with phosphorous. (*n* = 5).

Plant diversity values tended to be higher in NG samples compared with NGLP samples. However, the differences in means were not significant (paired *t*-test, *p* > 0.05) (Table 1). The NMDS scatter plot shows that samples from the two treatments form separate clusters (Figure 1). This observation is confirmed by PERMANOVA, which indicated that 36.9% of the variation observed was due to the effect of treatment (*p* < 0.05). The plant taxa that explained the most the variation were *Axonopus* (0.079), *Paspalum* (0.051), *Gaudinia* (0.058), and *Lotus* (0.057) (Figure S4). The relative abundance of all these genera, except for *Lotus*, was significantly different between the treatments (*p* < 0.05).

### 3.2. Effect of LP on Soil Microbial Diversity and Composition

Bacterial communities were dominated by the phyla Acidobacteriota (23.3% relative abundance, on average), Proteobacteria (20.7%), Verrucomicrobiota (15.7%), and Actinobacteria (10.2%) (Figure S5). There were no significant differences in relative abundance between treatments (paired *t*-test, *p* > 0.05). None of the diversity parameters analyzed (Richness, Shannon and Simpson index) showed significant differences between the two treatments (Table 1). The ordination result shows no clear arrangement of the sites according to treatment (PERMANOVA, *p* > 0.05) (Figure 1). Functional annotation of ASVs revealed twenty-two functions related to the energy source used by the bacterial taxa, as well as to different pathways of the C, N, and S cycles (Figure S6). There were no significant differences (*p* > 0.05) between NG and NGLP treatments in any of the functions according to the paired *t*-test.

Ascomycota was the most abundant fungal phylum, with a mean relative abundance of 46.9%, followed by Basidiomycota (29.7%) (Figure S7). A total of 8.6% of the reads could not be classified at the phylum level. As for bacteria, neither diversity (Table 1) nor community composition (Figure 1) showed significant differences between the two treatments. Nonetheless, the relative abundance of the fatty acid 18:1 ω9c, which has been described as diagnostic for fungi of the subphylum Mucoromycotina [44], was significantly lower (paired *t*-test, *p* < 0.05) in the NGLP treatment. This fact was somehow corroborated by fungal ITS amplicons sequencing, as the relative abundance of two ASVs (ASV51 and ASV239) assigned to the class Endogonomycetes (subphylum Mucoromycotina) were shown to be lower in the NGLP treatment than in the NG treatment (0.27% average in NGLP and 1.25% in NG) (paired *t*-test, *p* < 0.05). There were no significant differences (*p* > 0.05) between NG and NGLP treatments in any of the guilds or trophic modes (Figure S8) according to the paired *t*-test.

Microbial biomass and the relative abundance of most microbial groups, measured using specific PLFAs as biomarkers, showed no significant differences (Table 2). However, the relative abundance of AM Fungi was higher (paired *t*-test, *p* < 0.05) in the NG treatment. This difference, nevertheless, was not observed in the FUNGuild results (Figure S8). Additionally, the G- cy/pre ratio, used as an indicator of nutritional stress in bacterial communities, was found to be lower (paired *t*-test, *p* < 0.05) in the NG treatment than in the NGLP treatment (Figure S9).

Regarding *phoD* gene diversity in soils, the Richness and Shannon diversity index showed similar values in both treatments (Table 1). The *phoD*-harboring community does not appear to be arranged according to treatment in the NMDS plot PERMANOVA analysis (*p* > 0.05) (Figure 1). The dominant *phoD*-harboring genera were *Bradyrhizobium*, *Pseudomonas*, and *Bacillus* (Figure S10).

### 3.3. Effect of LP on Soil Microbial Respiration and Extracellular Enzyme Activities

In general, NGLP soils tended to have higher total enzyme activities than NG soils, but the differences were statistically significant (paired *t*-test *p* < 0.05) for BG activities only. However, specific activities (activity per unit of microbial biomass) of BG, NAG, and PHO enzymes were significantly higher (*p* < 0.05) in NGLP soils (Table 3). Both BG:(LAP + NAG) and BG:PHO ratios were lower than 0.5, but significantly higher (*p* < 0.05) in NGLP soils. No significant differences in microbial respiration were found between NG and NGLP soils (Table 3).

### 3.4. Relationships between Plant and Microbial Communities

Differences in plant community composition were significantly correlated with both bacterial and fungal community composition, but not with differences in *phoD*-harboring bacteria (Table 4). The correlation between plant and bacterial communities was stronger than the correlation between plant and fungal communities. Bacterial and fungal communities were also significantly correlated to each other and with the *phoD*-harboring community.

## 4. Discussion

Overseeding *L. subbiflorus*, in conjunction with P fertilization, modified the composition of the natural grassland plant communities in accordance with previous studies [11,45]. Both alien species and soil nutrient enrichment are known to impact the composition of plant communities [46,47]. For instance, fertilization disproportionally promotes fast-growing plants with more canopy cover and better access to light [48]. We note that although *Lotus* was among the top 10 plant genera that explained the difference between NG and NGLP, its relative abundance was not significantly different between management regimes, as previously reported [11]. This can be due to the fact that some herbivores seem to prefer to feed on nitrogen-fixing plants [49], reducing their growth. In contrast, LP did not affect the diversity of the plant community. Although the effect of plant diversity on productivity and resilience is very remarkable in grasslands with low levels of diversity (i.e., monoculture vs. mixed artificial grasslands) [50,51], this effect is less conclusive in high diversity systems [52].

We did not find support for our first hypothesis, as both bacterial and fungal populations showed no differences in alpha diversity between treatments. When considering grasslands across the globe, alpha diversities of plants and fungi were found to be uncorrelated [53]. However, in specific environments, positive correlation between bacterial and plant diversity has been found, for instance, as a response to N fertilization in a dry gradient of alpine grasslands [54]. Negative correlations have also been observed, for example, Piper et al. [55] determined that the invasion of *Bromus inermis* increased bacterial diversity while diminishing plant diversity. The lack of consistency in results may be due to differences in biogeography and pasture management systems between studies.

Similarly, microbial community composition did not differ between NG and NGLP soils. One possible explanation for this is that the soils in the NGLP treatments are reverting to the status before LP implementation, although the plant community still shows LP effect. Concomitantly, none of the functions inferred from FAPROTAX or FUNGuild showed significant differences between treatments, indicating a certain functional resilience of these soils to management. Previous works have shown that location can have strong effects on microbial communities [56,57,58], which can mask management effects.

The phylum Acidobacteria was the most abundant in our samples, most likely because the soils analyzed in our study had low pH. This phylum has been shown to be among the most abundant in soils worldwide [59], including grassland soils [60,61]. Unfortunately, although being very abundant, they are also among the less understood soil bacterial taxa, mainly as a result of their unculturability [62].

The genus *Candidatus* Udaeobacter was particularly abundant in our samples, which coincides with results from a study that analyzed 300 soil samples with pH values from 3.3 to 7.6 [63]. Currently, little is known about this bacterial genus; however, it is now considered ubiquitous in soil [64,65].

Interestingly, the fungi to bacteria ratio and the relative abundance of AM Fungi were higher in the NG treatment compared to the NGLP treatment. Soil with higher F:B are often considered as more sustainable in terms of plant productivity [66]. Fungi dominance is common in less managed environments as well as in natural soil ecosystems [67]. Moreover, fungal dominated soils are characteristic of soil under late succession stages of the plant community, with closed and slow nutrient cycles [12,68]. Thus, our results are in accordance with these expectations, as the NGLP soils received fertilization. Slower nutrient cycles, and therefore a signal of a more stable microbial community, can also be inferred from BG and NAG enzymatic activities, which were lower in NG than in NGLP soils.

The most abundant fungal phylum in our samples was Ascomycota, which is consistent with previous reports on the fungal community composition of grasslands, and is in accordance with the chemical nature of the litter produced in this biome [18]. Other types of vegetation such as forests, which produce more recalcitrant litter, tend to be dominated by Basidiomycota [69]. In grasslands, fungi are considered particularly reliant on plant communities and many studies have found fungal diversity to be correlated to plant diversity, even if bacteria are not [56,57,70]. This was, however, not observed in our study, which might indicate a higher resilience to environmental disturbances (i.e., NGLP treatment) of soil fungal communities in the Pampas grasslands.

It has been shown that P fertilization may decrease AMF diversity and abundance [71,72]. However, there are also reports indicating no significant differences under P fertilization, as previously observed in Uruguayan grasses [73]. We did not find differences either in alpha nor in beta diversity of fungi as a whole, but a decrease in the relative abundance of AM Fungi in NGLP soils was observed. Additionally, the subphylum Mucoromycotina might have been negatively affected by the NGLP treatment, as the relative abundance of the fatty acid 18:1 ω9c [44] was lower in such soils. The Mucoromycotina include arbuscule-forming fine root endophytes [74] other than the ubiquitous Glomeromycotinian AMF. However, there are currently a handful of studies on the ecology and distribution of these plant-symbiotic fungi.

Total extracellular enzyme activities in our study were similar to those observed in other grassland soils (e.g., Yang et al. [75]). The average BG:(NAG + LAP), BG:PHOS, and (NAG + LAP):PHOS ratios were much lower than the global mean values reported by Sinsabaugh et al. [76] for soils. Confirming our second hypothesis, there was an overall increase in enzyme activity in NGLP soils compared to NG soils. In particular, the significantly higher BG activity in NGLP soils could be related to higher plant litter inputs to soils and thus to the pool of cellulose-rich substrates available for decomposition. A positive association between BG activity and soil C content has been found across soil with very different pH values and management histories [77]. This can be explained, at least partially, by the fact that the presence of a substrate can stimulate an enzyme degrading the substrate (substrate stimulation model [78]).

Most studies suggest that soil organic C (energy) is the primary growth-limiting resource for soil microbial communities [79], although microbes with access to labile C in the fresh litter layer and rhizosphere may be nutrient limited [79]. In our study, according to the BG:(LAP + NAG) and BG:PHO ratios (both <0.5), N and P were the most limiting resources for microbial growth; similar to previous studies (e.g., Yang et al. [75]; Liu et al. 2023 [80]). The ecoenzymatic stoichiometry theory posits that microbes invest more in the production of extracellular enzymes able to acquire limiting resources [81]. Therefore, high microbial investments in N- and P-acquiring enzymes are suggestive of N and P co-limitation in NG and NGLP soils. Nevertheless, BG:(LAP + NAG) and BG:PHO ratios were significantly higher in NGLP soils (*p* < 0.05), as a result of enhanced BG activity, indicating that the microorganisms from NGLP soils invested more in C-acquiring enzymes than those from NG soils. NGLP soils are expected to have higher levels of N and P, due to the growth of N-fixing legumes and P fertilization and, therefore, be more productive, which might potentially increase C inputs to the soil via plant shoot and root litter production and release of root exudates [82]. Indeed, it was found that carbon-particulate organic matter (C-POM) stocks, a relatively short-lived form of soil carbon, are higher in NGLP soils [21]. More research is needed to understand the links between soil extracellular enzyme activity stoichiometry and soil nutrient stoichiometry in these soils.

We further found that BG, NAG, and PHOS specific enzyme activities were significantly increased in NGLP soils, indicating that NGLP soil microbial populations had a more pronounced enzyme activity. Compared with absolute enzyme activities, which indicate gross microbial activity, specific extracellular enzyme activities (activity per unit of microbial biomass C) represent the metabolic status of microbial communities [83,84]. Thus, evaluating specific extracellular enzyme activities may help to understand how microbial communities acclimatize to environmental changes and stresses. In our study, specific enzyme activities had a positive correlation with the G- cy/pre stress index, which were significantly higher (*p* < 0.05) in NGLP soils. A higher cyclopropyl to cyclopropyl precursors ratio is linked to decreased bacterial growth and an increase in carbon limitation [85,86]. This may suggest that microbes from NGLP soils try to adapt to intensified environmental pressures through enhancing the availability of C, N, and P. For instance, farmers usually apply higher stocking rates in NGLP paddocks [11].

## 5. Conclusions

It is important to stress that our study was carried out in sampling paddock pairs of privately owned and managed grasslands. Therefore, this constitutes an observational study where, on the one hand, many variables cannot be controlled but, on the other hand, it gives us the opportunity to measure the effect of management in biological populations under real-life practices, including the complexities of details in management. Legume overseeding together with phosphate fertilization clearly exerts an effect on plant community composition and this translates to changes in AM Fungi and extracellular enzyme activities. Overall, these results indicate that in the natural grasslands of the south-center region of Uruguay, which belongs to the Pampas biome, grassland management (i.e., P fertilization, sowing legumes) significantly influences the relative abundance of AM Fungi and several extracellular enzyme activities, which in turn could have consequences for the C, N, and P content of SOM. Thus, grassland management should be considered non-innocuous to the soil community and, as such, it should be carefully monitored to warrant sustainability.

## Figures and Tables

**Table 1:** Average richness and diversity parameters for each community analyzed. NG = natural grassland, NGLP = natural grassland overseeded with *Lotus subbiflorus* and fertilized with phosphorous. The standard deviation is shown in parenthesis. (*n* = 5).

Plant Community	Observed N	Shannon	Simpson
NG	57.6 (3.36)	2.84 (0.173)	0.898 (0.0266)
NGLP	45.4 (13.8)	2.52 (0.205)	0.875 (0.01)
Bacterial community			
NG	3278.0 (653.6)	7.18 (0.247)	0.998 (8.62 × 10^−4^)
NGLP	3511.8 (734.5)	7.22 (0.281)	0.997(1.69 × 10^−3^)
Fungal community			
NG	597.2 (42.8)	4.78 (0.444)	0.967 (0.0251)
NGLP	641.0 (55.3)	4.88 (0.342)	0.969 (0.0161)
*phoD* community			
NG	38.8 (5.1)	2.83 (0.164)	0.890 (0.012)
NGLP	24.8 (8.9)	2.46 (0.408)	0.864 (0.052)

**Table 2:** Phospholipid fatty acids analyses of the samples. Total microbial biomass is expressed as nanomoles of PLFAs per gram of dry soil. Abundances of microbial groups are expressed as the relative molar percentage of the sum of the diagnostic fatty acids after arcsine-square-root transformation. NG = natural grassland, NGLP = natural grassland overseeded with *Lotus subbiflorus* and fertilized with phosphorous. The standard deviation is shown in parenthesis. (*n* = 5).

	Biomass	Gram Negatives	Gram Positives	Actinobacteria	Fungi	AM Fungi	Fungi/ Bacteria	Gram+/Gram-
NG	123.0 (27.2)	0.730 (0.0143)	0.647 (0.0121)	0.365 (0.0119)	0.146 (0.0323)	0.132 (0.0153) **	4.92 (1.88) *	96.9 (3.54)
NGLP	121.0 (10.4)	0.740 (0.0146)	0.643 (0.0119)	0.366 (0.00791)	0.124 (0.0219)	0.111 (0.0241) **	3.49 (1.23) *	97.8 (2.96)

* Paired *t*-test *p* < 0.1. ** Paired *t*-test *p* < 0.05.

**Table 3:** Average extracellular enzymatic activities of the samples. AG = α-1,4-glucosidase, BG = β-1,4-glucosidase, CB = β-D-cellobiohydrolase, XYL = β-xylosidase, NAG = β-1,4-N-acetylglucosaminidase, LAP = L-leucine aminopeptidase, and PHO = acid phosphatase. CE = carbon related enzyme activities (AG + BG + CB + XYL), NE = nitrogen related enzyme activities (NAG + LAP). NG = natural grassland, NGLP = natural grassland overseeded with *Lotus subbiflorus* and fertilized with phosphorous. The standard deviation is shown in parenthesis. (*n =* 5).

	**AG**	**BG**	**CB**	**XYL**	**NAG**	**LAP**	**PHO**	**RESP**	**CE**	**NE**
NG	4.51 (1.53)	18.04 ** (3.18)	4.80 (2.23)	7.41 (2.72)	23.51 * (6.15)	14.27 (1.15)	129.09 (19.67)	0.69 (0.11)	34.76 (9.00)	37.78 * (7.19)
NGLP	5.45 (2.42)	25.85 ** (1.93)	7.33 (1.42)	10.65 (2.92)	31.98 * (4.71)	15.35 (1.58)	152.55 (21.88)	0.57 (0.11)	49.28 (6.58)	47.33 * (4.57)
NG ^1^	0.04 (0.01)	0.15 ** (0.03)	0.04 (0.02)	0.06 (0.03)	0.19 ** (0.04)	0.12 (0.02)	1.09 ** (0.28)			
NGLP ^1^	0.04 (0.02)	0.22 ** (0.03)	0.06 (0.01)	0.09 (0.03)	0.26 ** (0.02)	0.13 (0.02)	1.28 ** (0.27)			

* Paired *t*-test *p* < 0.1. ** Paired *t*-test *p* < 0.05. ^1^ Specific activities.

**Table 4:** Mantel test correlation (Spearman) results between the communities analyzed. (*n =* 5).

	Plant	Bacteria	Fungi	*phoD*
Plant	-----	0.4313 **	0.318 *	0.1753
Bacteria		----	0.7175 ***	0.621 ***
Fungi			----	0.5717 ***
*phoD*				----

* *p* < 0.1. ** *p* < 0.05. *** *p* < 0.05.

## Data Availability

The raw DNA sequences files used in this study are deposited in the (Sequence Read Archive) SRA of the NCBI under the project number PRJNA961077.

## Supplementary Materials

The following supporting information can be downloaded at: https://www.mdpi.com/article/10.3390/microorganisms11061383/s1, Figure S1. Relative abundances of the main plant classes in each site. Figure S2. Relative abundances of the main plant families in each site. Figure S3. Relative abundances of the main plant genera in each site. Figure S4. Main plant genera that explained most of the variation observed due to treatment effect on plant community. Figure S5. Relative abundances of the main bacterial phyla in each site. Figure S6. Heatmap representing abundances of functions and metabolisms inferred from the 16S rRNA gene data for each site. Figure S7. Relative abundances of the main fungal phyla in each site. Figure S8. Heatmap representing abundances of fungal trophic modes and guilds inferred from the ITS data for each site. Figure S9. Boxplot of G- cy/pre ratio, indicative of nutritional stress for each treatment. Figure S10. Relative abundances of the main *phoD*-harboring genera in each site. Table S1. Soil characteristic of the sites sampled.
