# Supplementary material for: Legume Overseeding and P Fertilization Increases Microbial Activity and Decreases the Relative Abundance of AM Fungi in Pampas Natural Pastures
Source: Microorganisms. 2023 May 24;11(6):1383. doi: 10.3390/microorganisms11061383 (PMC10302908; doi:10.3390/microorganisms11061383)
Supplement: Supplementary file 1 [file microorganisms-11-01383-s001.zip › Supplementary material legends.pdf]

Figure S1. Relative abundances of the main plant classes in each site. NG= natural grassland, NGLP= natural grassland overseeded with *Lotus subbiflorus* and fertilized with phosphorous.

Figure S2. Relative abundances of the main plant families in each site. NG= natural grassland, NGLP= natural grassland overseeded with *Lotus subbiflorus* and fertilized with phosphorous.

Figure S3. Relative abundances of the main plant genera in each site. NG= natural grassland, NGLP= natural grassland overseeded with *Lotus subbiflorus* and fertilized with phosphorous.

Figure S4. Main plant genera that explained most of the variation observed due to treatment effect on plant community. The 10 most explanatory genera are shown.

Figure S5. Relative abundances of the main bacterial phyla in each site. NG= natural grassland, NGLP= natural grassland overseeded with *Lotus subbiflorus* and fertilized with phosphorous.

Figure S6. Heatmap representing abundances of functions and metabolisms inferred from the 16S rRNA gene data for each site. NG= natural grassland, NGLP= natural grassland overseeded with *Lotus subbiflorus* and fertilized with phosphorous.

Figure S7. Relative abundances of the main fungal phyla in each site. NG= natural grassland, NGLP= natural grassland overseeded with *Lotus subbiflorus* and fertilized with phosphorous.

Figure S8. Heatmap representing abundances of fungal trophic modes and guilds inferred from the ITS data for each site. NG= natural grassland, NGLP= natural grassland overseeded with *Lotus subbiflorus* and fertilized with phosphorous.

Figure S9. Boxplot of G- cy /pre ratio, indicative of nutritional stress for each treatment. NG= natural grassland, NGLP= natural grassland overseeded with *Lotus subbiflorus* and fertilized with phosphorous.

Figure S10. Relative abundances of the main *phoD*-harbouring genera in each site. NG= natural grassland, NGLP= natural grassland overseeded with *Lotus subbiflorus* and fertilized with phosphorous.
