# Supplementary material for: Legume Overseeding and P Fertilization Increases Microbial Activity and Decreases the Relative Abundance of AM Fungi in Pampas Natural Pastures
Source: Microorganisms. 2023 May 24;11(6):1383. doi: 10.3390/microorganisms11061383 (PMC10302908; doi:10.3390/microorganisms11061383)
Supplement: Supplementary file 1 [file microorganisms-11-01383-s001.zip › Table S1.pdf]

**Table S1**. Soil characteristic of the sites sampled. Basic physicochemical data, year of LP implementation and location of the sites are shown.

| Site and management | Year when the LP was established | Cumulative P fertilization (kg/ha $P_2O_5$) | pH | EC[1] dS/m | P Bray 1 mg/kg | EA[2] $cmol_c/kg$ | Coordinates |
|---|---|---|---|---|---|---|---|
| SUL2 - NG | - | - | 5.1 | 0.103 | 1.7 | 0.20 | 33°52'23"S; 55°35'21"W |
| SUL2 - NGLP | 1994 | 695 | 5.1 | 0.123 | 4.6 | 0.15 | 33°52'23"S; 55°35'21"W |
| SUL4 - NG | - | - | 5.1 | 0.108 | 1.1 | 0.22 | 33°52'4"S; 55°35'8"W |
| SUL4 -NGLP | 1994 | 695 | 5.2 | 0.143 | 3.7 | 0.15 | 33°52'4"S; 55°35'8"W |
| SUL5 - NG | - | - | 5.1 | 0.056 | 1.0 | 0.18 | 33°51'41"S; 55°34'51"W |
| SUL5 - NGLP | 2005 | 463 | 5.2 | 0.134 | 0.9 | 0.24 | 33°51'41"S; 55°34'51"W |
| SCFR - NG | - | - | 5.3 | 0.133 | 1.1 | 0.08 | 33°51'23"S; 55°21'4"W |
| SCFR - NGLP | 2010 | 139 | 5.2 | 0.118 | 10.3 | 0.09 | 33°51'23"S; 55°21'4"W |
| SCFO - NG | - | - | 5.2 | 0.152 | 2.8 | 0.11 | 33°51'29"S; 55°20'25"W |
| SCFO - NGLP | 2003 | 212 | 5.0 | 0.144 | 2.0 | 0.13 | 33°51'29"S; 55°20'25"W |

[1]EC= electrical conductivity

[2]EA= Exchangeable acidity
