# Supplementary figures and images for: Legume Overseeding and P Fertilization Increases Microbial Activity and Decreases the Relative Abundance of AM Fungi in Pampas Natural Pastures
Source: Microorganisms. 2023 May 24;11(6):1383. doi: 10.3390/microorganisms11061383 (PMC10302908; doi:10.3390/microorganisms11061383)

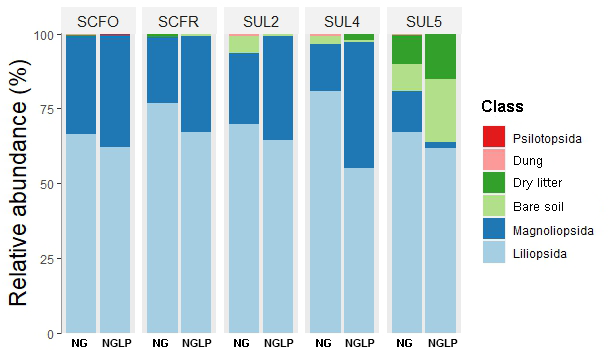

Supplement: Supplementary file 1 [file microorganisms-11-01383-s001.zip › Figure S1.tif]

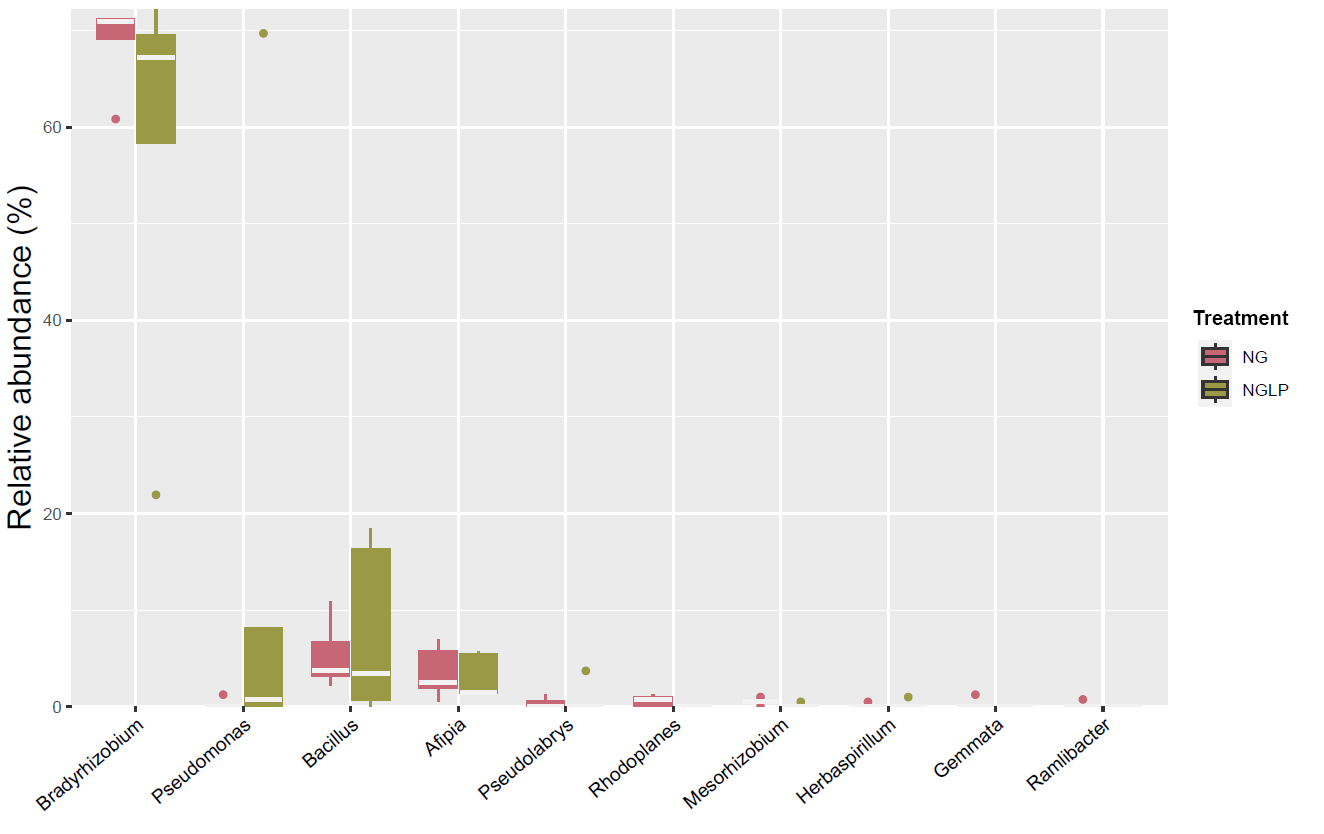

Supplement: Supplementary file 1 [file microorganisms-11-01383-s001.zip › Figure S10.tif]

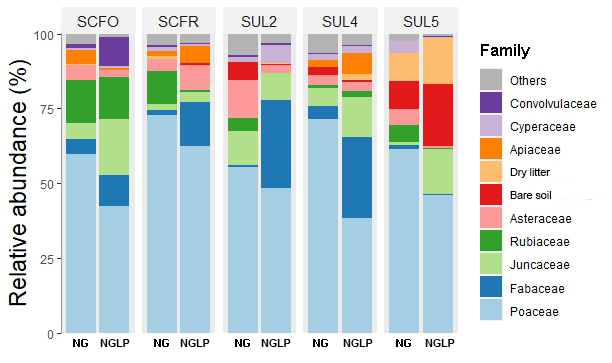

Supplement: Supplementary file 1 [file microorganisms-11-01383-s001.zip › Figure S2.tif]

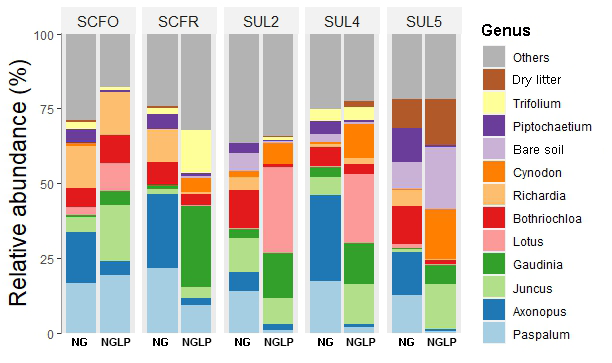

Supplement: Supplementary file 1 [file microorganisms-11-01383-s001.zip › Figure S3.tif]

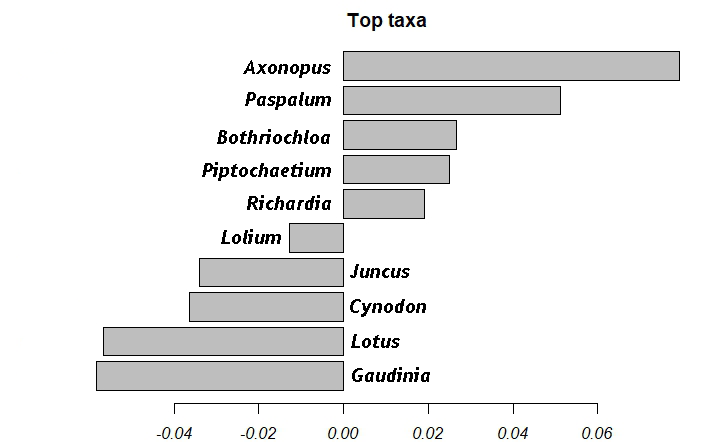

Supplement: Supplementary file 1 [file microorganisms-11-01383-s001.zip › Figure S4.tif]

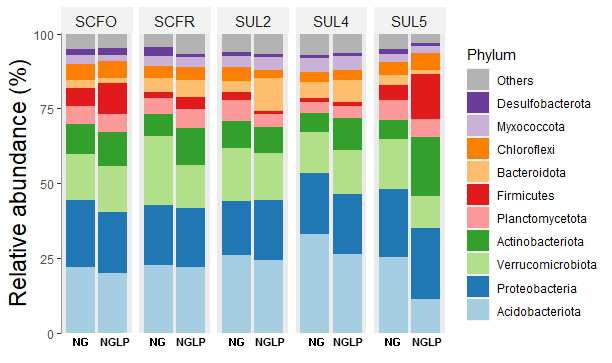

Supplement: Supplementary file 1 [file microorganisms-11-01383-s001.zip › Figure S5.tif]

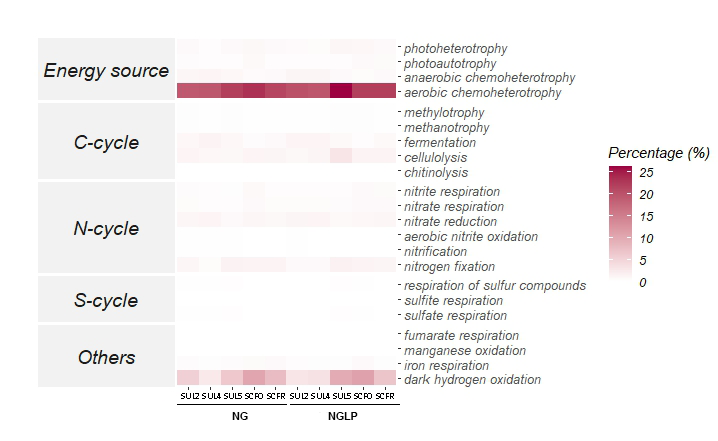

Supplement: Supplementary file 1 [file microorganisms-11-01383-s001.zip › Figure S6.tif]

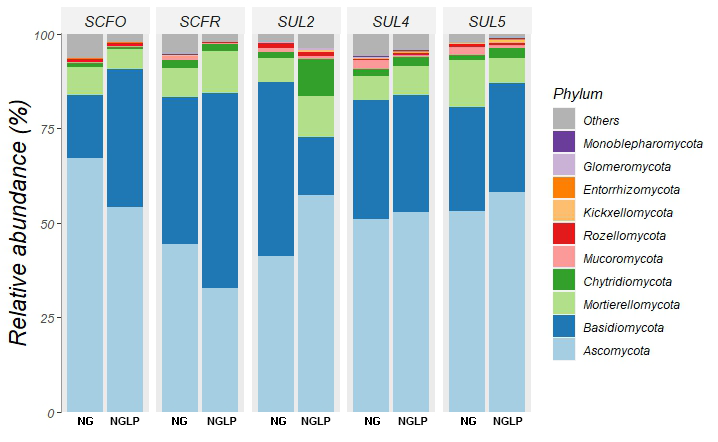

Supplement: Supplementary file 1 [file microorganisms-11-01383-s001.zip › Figure S7.tif]

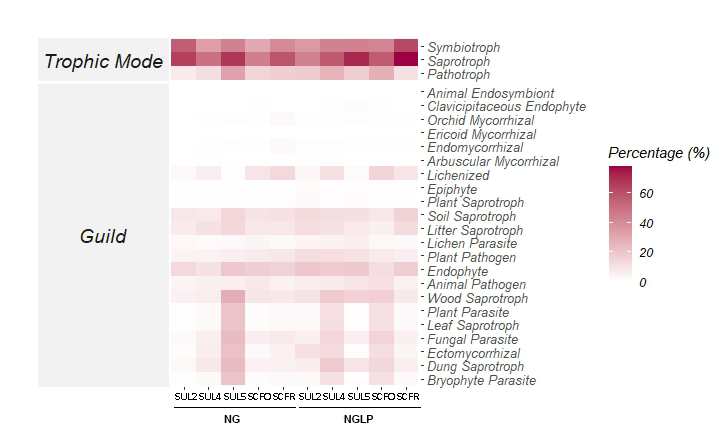

Supplement: Supplementary file 1 [file microorganisms-11-01383-s001.zip › Figure S8.tif]

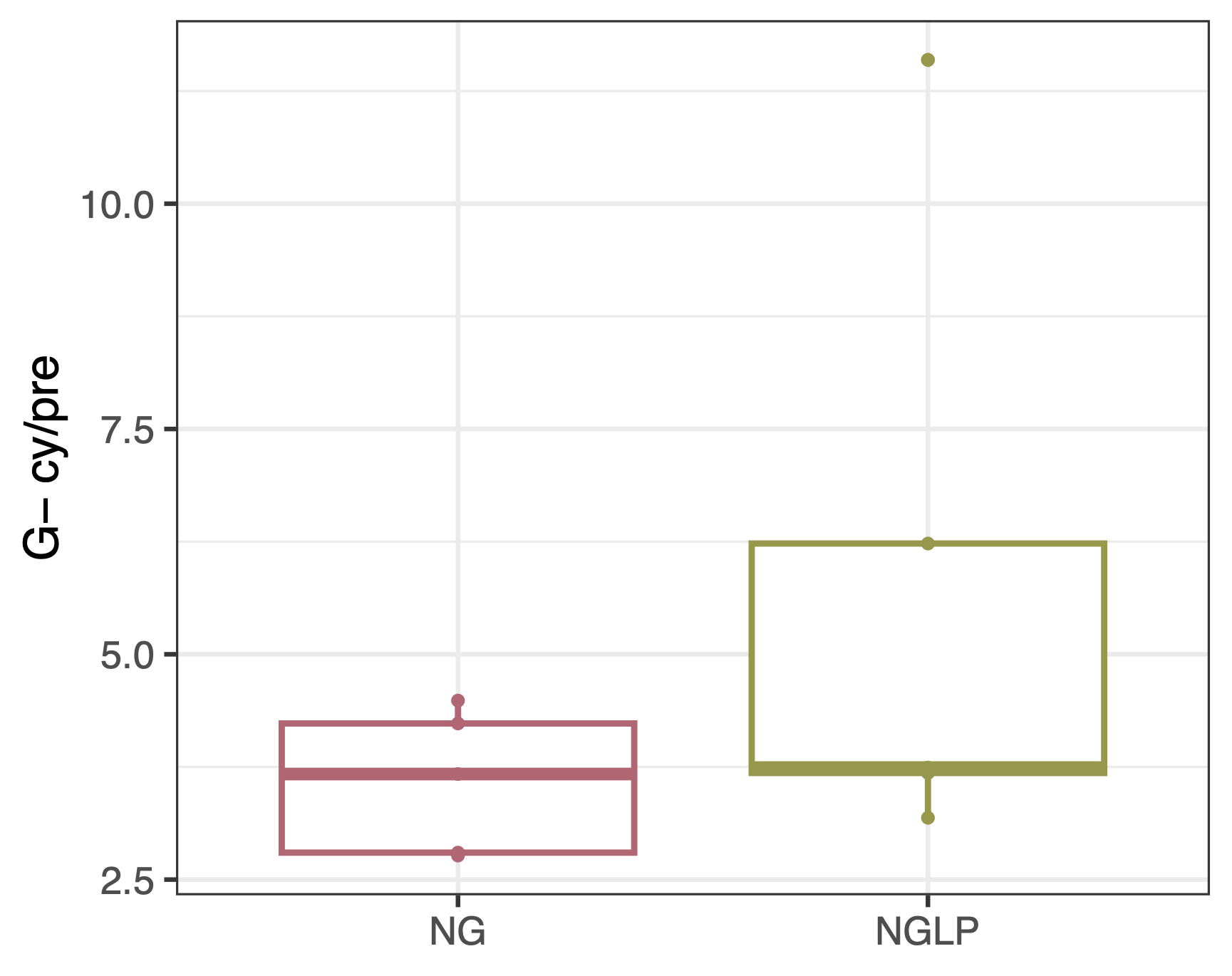

Supplement: Supplementary file 1 [file microorganisms-11-01383-s001.zip › Figure S9.tif]
